# Challenges and Prospects for Surrogacy in Iran as a Pioneer Islamic Country in this Field

**DOI:** 10.34172/aim.33920

**Published:** 2025-04-01

**Authors:** Mohammad Haddadi, Sedigheh Hantoushzadeh, Parisa Hajari, Roya Rashid Pouraie, Mir Yasha Hadiani, Gholam Reza Habibi, Nasim Eshraghi, Marjan Ghaemi

**Affiliations:** ^1^Vali-E-Asr Reproductive Health Research Center, Family Health Research Institute, Tehran University of Medical Sciences, Tehran, Iran; ^2^Criminal Science and Criminology Department, Faculty of Law and Political Science, University of Tehran, Tehran, Iran

Infertility rates are increasing globally^1^; according to a recent analysis, the overall prevalence of infertility in Iran is around 8%, which is rising slowly.^2^ In addition to the fundamental right to decide the number, timing, and spacing of children for each individual and couple, as stated in human rights documents,^3^ there are many social, economic, and psychological consequences for men and women who experience infertility, including depression, anxiety, abuse, and divorce.^4^ Surrogacy is one of the treatment choices for infertile couples, due to absolute uterine factor or for women who are unable to become pregnant due to their physical condition.^5^ Many countries have prohibited surrogacy in their laws, such as France, and Turkey.^6,7^ However, in Iran, it has been practiced since 2003 after the Embryo Donation Action by a broad interpretation of the law. Nevertheless, some medical, ethical, legal, and religious concerns still exist.

 One of the main concerns for surrogate mothers is lack of consideration for their basic health condition and the need for comprehensive medical and psychological screening to ensure they are capable of carrying a pregnancy to term. This is especially important given the high rate of multiple pregnancies associated with surrogacy, which can increase the risk of complications for both the surrogate mother and the fetus.^8^ Pregnancy-related chronic diseases, like peripartum cardiomyopathy, may require immediate termination, posing challenges for all parties involved. Biological parents might refuse to cover the surrogate’s medical expenses, arguing payments were intended for childbirth. Lack of specific provisions in surrogacy contracts exacerbates disputes, highlighting the need for clear legal guidelines.^9^ Iran’s population is predominantly Muslim, primarily Shi’a, with a minority of Sunnis. Despite sharing the Qur’an as their holy book, differences arise from distinct historical, ethnic, political, and social developments. Sunni scholars generally permit assisted reproductive technologies (ART) if limited to the biological parents’ gametes. However, practices involving third parties, such as embryo donation and surrogacy, are deemed morally corrupt (“*fasad*”).^10^

 Shi’a clerics in Iran hold varied views on assisted reproduction. In 2000, the Research Center of the Islamic Legislative Assembly reviewed major Shi’a scholars’ opinions on third-party eggs, sperm, embryo donation, and surrogacy. While some clerics advocate caution, most religious decrees adopt a more flexible stance, generally permitting these practices.^11^ Ayatollah Khamenei, Iran’s political and ecclesiastical authority, declared that surrogacy is permitted and a child born through ART is considered attached to the biological parents (owner of the sperm and egg).^12^ Ayatollah Sistani, a prominent Shi’a authority, permits artificial insemination using third-party gametes and surrogacy. However, he offers no clear rulings on inheritance and motherhood for children born through ART, instead recommending contractual agreements between biological parents and surrogates to address these matters.^13^

 The maternal relationship between a surrogate mother and the child remains debated in Islam. In Iran, surrogates have no legal rights over the child, who is registered under the intended parents. Whether the surrogate is considered a second mother, as suggested by Qur’anic verses, is unresolved. No Fatwa addresses the child’s relationship with the surrogate’s other children or potential marriage eligibility. Additionally, while Shi’a authorities discourage surrogacy for profit, they support reasonable compensation for surrogates.^14^

 Commercial surrogacy is an important issue that requires consideration. It is accepted that reimbursements must be paid to those who provide eggs, sperm, or embryos. However, the ethics of offering these services solely for financial purposes have been a matter of protracted debate.^15^ In Iran, there are significant concerns about commercial surrogacy. Aramesh et al have declared that commercial surrogacy is considered unethical due to the exploitation of human beings, particularly vulnerable women in developing countries, and the commodification of what should not be bought or sold based on human dignity.^16^

 Surrogacy agreements between the surrogate mother and the intended parents can be performed and registered in one of the branches of the Deeds and Properties Registration Organization, with the presence of the parties’ lawyers, unless they significantly contradict Islamic law.^17,18^ The document is typically prepared based on a lease contract of the personal lease type, whereby the lessee undertakes the responsibility for the pregnancy, care, delivery, and upbringing of the fetus for a certain period.^19^

 Some studies discuss the effects of dissolving surrogacy contracts. For example, Safari et al have declared that dissolving the surrogacy contract during pregnancy, both before and after the transfer of the embryo, is in line with the general rules of contracts in Iran.^20^ Additionally, the parties involved may agree to compensate each other for damages arising from the surrogacy agreement, such as reducing compensation or using a surrogate mother’s guarantee against genetic parents. However, the term “nonliability” is considered illegal when it pertains to the life of a human being.^21^ Furthermore, the absence of clear laws on commercial surrogacy has fueled a costly black market. Economic struggles in low-income groups force some into surrogacy, often involving mothers over 35, increasing pregnancy risks.^22^ Nevertheless, no clear laws exist to distinguish the surrogate mothers’ rights when facing pregnancy complications, which may often result in the termination of the pregnancy.

 In all these situations, the law does not clarify the financial and legal duties of the intended parents toward the surrogate mother, nor does it address the rights of the surrogate mother in case she faces a severe risk during and after pregnancy due to pregnancy complications.

 Despite being a pioneer in surrogacy legislation, Iran lacks comprehensive laws, particularly regarding surrogate mothers’ rights and commercial surrogacy, leading to potential abuses. The rapid growth of surrogacy raises ethical and legal challenges, especially in religious contexts. While Iran has made notable progress, further improvements require clear legal frameworks, public awareness, and enhanced medical screening to ensure safety and ethical standards. Addressing unresolved issues, such as third-party parental roles, health rights, and commercial practices, is essential to protect human rights and uphold dignity for all involved.

## References

[1] Mascarenhas MN, Flaxman SR, Boerma T, Vanderpoel S, Stevens GA (2012). National, regional, and global trends in infertility prevalence since 1990: a systematic analysis of 277 health surveys. PLoS Med.

[2] Saei Ghare Naz M, Ozgoli G, Sayehmiri K (2020). Prevalence of infertility in Iran: a systematic review and meta-analysis. Urol J.

[3] OHCHR. Convention on the Elimination of All Forms of Discrimination against Women. New York: OHCHR; 1979. Available from: https://www.ohchr.org/sites/default/files/cedaw.pdf. Accessed April 17, 2023.

[4] Rouchou B (2013). Consequences of infertility in developing countries. Perspect Public Health.

[5] Söderström-Anttila V, Wennerholm UB, Loft A, Pinborg A, Aittomäki K, Romundstad LB (2016). Surrogacy: outcomes for surrogate mothers, children and the resulting families-a systematic review. Hum Reprod Update.

[6] Madanamoothoo A. Surrogacy under French law: ethical, medical, and legal issues. In: Beran RG, ed. Legal and Forensic Medicine. Berlin, Heidelberg: Springer; 2013. p. 1545-59. 10.1007/978-3-642-32338-6_102.

[7] Ebrahimi A, Ghodrati F (2025). Comparative investigation of surrogacy laws in Asian Islamic countries: a narrative review. J Midwifery Reprod Health.

[8] Hosseini S, Asghari E, Behmanesh F (2014). Surrogacy in Iran: the medical costs of multiple pregnancies. Arch Iran Med.

[9] Fazeli Z, Abedi P, Abedian Z. Ethical and legal aspects of surrogacy from Iranian specialists’ viewpoints. J Med Ethics Hist Med 2015;8(9).

[10] Inhorn MC (2006). Making Muslim babies: IVF and gamete donation in Sunni versus Shi’a Islam. Cult Med Psychiatry.

[11] Behjati-Ardakani Z, Karoubi MT, Milanifar A, Masrouri R, Akhondi MM (2015). Embryo donation in Iranian legal system: a critical review. J Reprod Infertil.

[12] Khamenei A. KHAMENEI.IR. Available from: https://farsi.khamenei.ir/treatise-index. Accessed June 26, 2023.

[13] Medical Issues - Question & Answer. The Official Website of the Office of His Eminence Al-Sayyid Ali Al-Husseini Al-Sistani. Available from: https://www.sistani.org/english/qa/01249/. Accessed May 28, 2023.

[14] Heidari H. Jurisprudential and legal analysis of the effects of artificial insemination through surrogacu. J Law Res Ghanonyar 2020;2(5). [Persian].

[15] Brandão P, Garrido N (2022). Commercial surrogacy: an overview. Rev Bras Ginecol Obstet.

[16] Aramesh K. Ethical assessment of monetary relationship in surrogacy. Med J Reprod Infertil 2008;9(1):36-42. [Persian].

[17] Civil Code of the Islamic Republic of Iran. Published online December 14, 1935.

[18] Islamic Parliament Research Center. Available from: https://rc.majlis.ir/fa/law/show/93943. Accessed April 19, 2023.

[19] Qasemzadeh SM. Surrogacy contracts in the Iranian law. J Reprod Infertil 2008;9(2):182-94. [Persian].

[20] Safari M, Shahini M (2020). The effects of dissolving the surrogacy contracts. Legal Research Quarterly.

[21] Yadollahi Baghlouei A, Asadinejad SM. The research of contractual liability of surrogate mother against genetic parents. Med Law J 2013;7(25):125-56. [Persian].

[22] Dashtizadeh P, Mazloom Rahni A, Rajabzadeh A (2019). Legal and ethical challenges of surrogacy contracts in Iranian judicial and medical system. Health Spiritual Med Ethics.

